# Beyond Diagnostic Cut-Offs: Associations Between the sFlt-1/PlGF Ratio and Perinatal Outcomes in Low-Risk Term Pregnancies

**DOI:** 10.3390/jcm15124679

**Published:** 2026-06-16

**Authors:** Karolina Bednarz, Maisa Manasar-Dyrbuś, Marcin Sadłocha, Magdalena Bednarek-Jędrzejek, Rafał Stojko, Jakub Staniczek

**Affiliations:** 1 Department of Gynecology, Obstetrics and Gynecological Oncology, Medical University of Silesia, 40-211 Katowice, Polandmsadlocha@sum.edu.pl (M.S.);; 2 Department of Gynecology and Obstetrics, Pomeranian Medical University, 70-111 Szczecin, Poland

**Keywords:** sFlt-1/PlGF ratio, sFlt-1, PlGF, pregnancy complications

## Abstract

**Background/Objectives**: The soluble fms-like tyrosine kinase-1 (sFlt-1) to placental growth factor (PlGF) ratio is an established biomarker in the diagnosis of preeclampsia; however, its significance outside overt hypertensive disorders of pregnancy remains unclear. Emerging evidence suggests that angiogenic imbalance may reflect subclinical placental dysfunction even in otherwise low-risk pregnancies. To investigate associations between the sFlt-1/PlGF ratio and maternal and neonatal outcomes in a low-risk term obstetric population, beyond established diagnostic cut-offs. **Methods**: This prospective cohort study included 87 women with singleton term pregnancies. Serum sFlt-1 and PlGF concentrations were measured at hospital admission before delivery, and the sFlt-1/PlGF ratio was calculated. The primary outcome was estimated blood loss at delivery. Secondary maternal outcomes included postpartum hemoglobin decline, uterine atony, and fibrinogen concentration. Neonatal outcomes included birthweight, umbilical artery pH, and bilirubin concentration. Multivariable regression models were used to evaluate associations between the ln-transformed sFlt-1/PlGF ratio and outcomes after adjustment for prespecified maternal and obstetric covariates. **Results**: Each doubling of the sFlt-1/PlGF ratio was associated with greater estimated peripartum blood loss (+78.0 mL, 95% CI 42.1–113.9; *p* < 0.001), a larger postpartum hemoglobin decline (+0.078 g/dL, 95% CI 0.008–0.148; *p* = 0.030), lower fibrinogen concentration (−20.7 mg/dL, 95% CI −30.5 to −10.9; *p* < 0.001), and lower neonatal birthweight (−64.6 g, 95% CI −102.0 to −27.2; *p* = 0.001). No significant associations were observed for uterine atony, premature rupture of membranes, or umbilical artery pulsatility index above the 75th centile. **Conclusions**: In low-risk term pregnancies, higher sFlt-1/PlGF ratios were associated with greater estimated peripartum blood loss, lower fibrinogen concentrations, and lower neonatal birthweight. These findings support the hypothesis that variation in angiogenic balance may reflect subclinical placental dysfunction even in apparently uncomplicated pregnancies. Further prospective studies are needed to validate these exploratory observations and determine their clinical relevance.

## 1. Introduction

### 1.1. Background

Placental vascular architecture plays a central role in maintaining adequate uteroplacental blood flow and supporting optimal fetal growth. This process depends on a tightly regulated balance between proangiogenic and antiangiogenic factors, which modulate vascular remodeling, endothelial function, and placental blood flow throughout gestation [1,2].

Placental growth factor (PlGF), a member of the vascular endothelial growth factor (VEGF) family, is predominantly expressed in the placenta. It contributes to physiological placental vascular development and maternal vascular adaptation during gestation [3]. In contrast, sFlt-1, a circulating soluble isoform of VEGF receptor-1 (Flt-1), functions as a decoy receptor that binds VEGF and PlGF, thereby reducing their bioavailability and attenuating downstream proangiogenic signaling in the maternal circulation [4]. During normal pregnancy, circulating PlGF and sFlt-1 concentrations change dynamically with advancing gestation, resulting in a progressive increase in the sFlt-1/PlGF ratio toward term [5,6].

Placental hypoxia and impaired placental development have been associated with increased release of antiangiogenic mediators, particularly sFlt-1, leading to an antiangiogenic state in the maternal circulation. Such imbalance has been linked to adverse pregnancy outcomes, including fetal growth abnormalities and complications associated with placental dysfunction [1,7,8].

The opposing biological effects of sFlt-1 and PlGF on placental angiogenesis make their combined assessment a more integrative measure of angiogenic imbalance than either biomarker alone. Accordingly, the sFlt-1/PlGF ratio has been widely used in clinical practice, particularly in the context of hypertensive disorders of pregnancy, where predefined cut-off values enable diagnosis, risk stratification, and exclusion of preeclampsia with high predictive accuracy [9,10,11,12]. However, outside these high-risk settings, the clinical implications of varying sFlt-1/PlGF levels remain less clearly defined, particularly about perinatal and neonatal outcomes in low-risk, term pregnancies.

To date, most studies of the sFlt-1/PlGF ratio have focused on high-risk pregnancies complicated by preeclampsia, fetal growth restriction, or other manifestations of placental dysfunction, primarily evaluating the biomarker within established diagnostic cut-off frameworks [13,14,15,16,17]. Low-risk pregnancies, in contrast, provide a clinical model to explore variations in angiogenic balance in the absence of overt placental pathology and to examine whether such variation has functional relevance at delivery.

Data regarding the relationship between the sFlt-1/PlGF ratio and intrapartum events, postpartum maternal condition, and neonatal outcomes in low-risk term pregnancies remain limited. Therefore, this study aimed to evaluate the full spectrum of sFlt-1/PlGF values in a consecutive cohort of low-risk women delivering at term and to assess their associations with maternal and neonatal outcomes.

### 1.2. Objective

This study evaluates the sFlt-1/PlGF ratio as a continuous variable in low-risk term pregnancies, moving beyond predefined diagnostic cut-offs. We aimed to examine associations between angiogenic balance and intrapartum course, maternal postpartum condition, and neonatal outcomes, and to characterize how perinatal risk varies across the full range of sFlt-1/PlGF values.

## 2. Methods

### 2.1. Study Design

This was a prospective, non-interventional study involving sample and data collection. The study protocol was approved by the Review Board of the Chair and Department of Gynecology, Obstetrics, and Gynecological Oncology at the Medical University of Silesia in Katowice (approval code: 19/2022; approved on 15 August 2022) and by the Bioethics Committee of the Medical University of Silesia in Katowice (approval number: BNW/NWN/0052/KB1/36/23; approved on 24 April 2023). The study was reported in accordance with the Strengthening the Reporting of Observational Studies in Epidemiology (STROBE) [18].

### 2.2. Settings

From 1 October 2024, to 31 December 2024, all patients meeting the predefined inclusion criteria were consecutively enrolled. The study was conducted at the Clinical Department of Gynecology and Obstetrics with a Subdepartment of Pediatric and Adolescent Gynecology, Bonifratres Medical Centre—Brothers Hospitallers of St. John of God, Katowice, Poland. Data analysis was performed by researchers from the Chair and Department of Gynecology, Obstetrics, and Gynecological Oncology at the Medical University of Silesia in Katowice, Poland.

### 2.3. Participants

Women with term singleton pregnancies were eligible for inclusion. Term pregnancy was defined as delivery at ≥37 + 0 weeks of gestation. Only women with uncomplicated pregnancies throughout the antenatal period were included, representing a cohort of healthy, low-risk obstetric patients.

Both vaginal and cesarean deliveries were included. The study population was ethnically homogeneous and consisted exclusively of women of White ethnicity. All participants met the predefined eligibility criteria and provided written informed consent prior to participation.

Exclusion criteria included instrumental delivery and planned elective induction of labor. However, augmentation of spontaneous labor was permitted when clinically indicated. In such cases, oxytocin was administered via an infusion pump according to standard clinical protocols, most commonly in cases of premature rupture of membranes (PROM). Maternal conditions known to influence angiogenic balance or perinatal outcomes were also excluded, including chronic kidney disease, chronic hypertension, gestational hypertension, preeclampsia, pregestational or gestational diabetes mellitus, and antiphospholipid syndrome. Pregnancies classified as high risk for preeclampsia based on first-trimester screening were also excluded. Women receiving acetylsalicylic acid for preeclampsia prophylaxis were not eligible for inclusion.

Pregnancies complicated by fetal growth restriction, defined according to the INTERGROWTH-21st standards, were excluded [19]. Fetuses with major congenital anomalies or chromosomal abnormalities were also excluded.

To ensure temporal consistency between biomarker assessment and clinical outcomes, women who delivered more than 7 days after measurement of the sFlt-1/PlGF ratio were excluded. Participants were not selected based on angiogenic marker values.

### 2.4. Variable

Definitions of the exposure, primary outcomes, secondary variables, and covariates are provided in Table 1.

### 2.5. Data Source

Maternal blood samples were obtained at hospital admission, before delivery, as part of routine clinical evaluation. Samples were centrifuged and stored at −40 °C until analysis. Maternal serum concentrations of sFlt-1 and PlGF were subsequently measured using kits by Thermo Fisher Scientific, Waltham, MA, USA, and the sFlt-1/PlGF ratio was calculated thereafter. Biomarker measurements were performed in batch after completion of participant recruitment and after all deliveries had occurred.

Obstetric ultrasound examinations, including fetal biometry and assessment of amniotic fluid volume, were performed in accordance with standard diagnostic protocols [21]. Maternal and neonatal outcomes were prospectively recorded according to a predefined study protocol. Complete blood count, fibrinogen concentration, and coagulation parameters were assessed at hospital admission before delivery. Postpartum maternal hemoglobin concentration was assessed during the first postpartum day, approximately 24 h after delivery. Neonatal total bilirubin concentration was measured after birth and reassessed on the second day of life.

### 2.6. Bias

Potential bias inherent to the observational design was addressed through prespecified eligibility criteria and standardized data collection.

### 2.7. Study Size and Participants Flow

During the study period, 100 pregnant women were assessed for eligibility. Thirteen women were excluded, including eight who delivered more than 7 days after sFlt-1/PlGF measurement and five who met predefined exclusion criteria. The final study cohort, therefore, comprised 87 women, who were included in the primary analysis.

### 2.8. Statistical Methods

Continuous variables were assessed for distributional characteristics and are presented as medians with interquartile ranges, while categorical variables are reported as counts and percentages. The sFlt-1/PlGF ratio was natural log-transformed (ln) prior to analysis due to right skewness. Log-transformation was applied for statistical modeling and quartile definition, whereas outcomes and between-quartile contrasts are presented in the original clinical units to facilitate interpretation. For clarity, a doubling refers to a two-fold (100%) increase in the sFlt-1/PlGF ratio (e.g., from 10 to 20 or from 20 to 40).

Associations between the sFlt-1/PlGF ratio and maternal and neonatal outcomes were evaluated using multivariable linear regression for continuous outcomes and logistic regression for binary outcomes. Given the relatively small sample size, the number of covariates included in the models was intentionally limited to reduce the risk of model overfitting. Covariates were selected a priori based on clinical relevance. To facilitate clinical interpretability and to explore potential nonlinear associations, a complementary quartile-based analysis was performed by comparing the highest and lowest quartiles of the log-transformed sFlt-1/PlGF ratio. Analyses stratified by mode of delivery were conducted for descriptive and exploratory purposes only; all primary analyses were performed in the overall cohort, in line with the study objective. Quartile-based outcome distributions were visualized using violin plots.

As part of exploratory analyses, the association between the sFlt-1/PlGF ratio and amniotic fluid volume was evaluated using Spearman’s rank correlation coefficient (ρ) due to non-normal distributions. Analyses were performed on continuous variables without adjustment for confounders, as they were intended for hypothesis generation. Given the exploratory design and modest effect size, this association was not included in multivariable models.

No missing data were present for variables included in the analyses; therefore, all enrolled participants were included in the regression models.

All statistical tests were two-sided, and *p*-values < 0.05 were considered statistically significant. All analyses were conducted using Python version 3.11 (Python Software Foundation, Wilmington, DE, USA).

## 3. Results

### 3.1. Study Population

Baseline maternal, angiogenic, obstetric, and neonatal characteristics of the study cohort are summarized in Table 2a,b.

### 3.2. Binary Outcomes

Uterine atony occurred in 12 women (13.8%), premature rupture of membranes (PROM) in 22 women (25.3%), and umbilical artery PI > 75th centile in 17 women (19.5%).

For binary outcomes, multivariable logistic regression models adjusted for maternal age, body mass index, parity, and gestational age at delivery were applied. The sFlt-1/PlGF ratio was not significantly associated with the occurrence of PROM, uterine atony or umbilical artery pulsatility index above the 75th centile in adjusted analyses.

### 3.3. Continuous Outcomes

In multivariable linear regression models adjusted for maternal age, body mass index, parity, and gestational age at delivery, the sFlt-1/PlGF ratio was significantly associated with maternal bleeding-related outcomes and selected neonatal parameters.

A higher sFlt-1/PlGF ratio was independently associated with a greater peripartum decrease in maternal hemoglobin concentration (ΔHb). Each doubling of the sFlt-1/PlGF ratio (i.e., a two-fold increase, e.g., from 10 to 20) was associated with an increase in ΔHb of 0.078 g/dL (*p* = 0.030). The strongest association was observed for estimated blood loss at delivery. Each doubling of the sFlt-1/PlGF ratio was associated with an estimated increase in blood loss of 78.0 mL (*p* < 0.001).

Higher sFlt-1/PlGF values were also associated with lower absolute maternal hemoglobin concentrations after delivery. Each doubling of the ratio was associated with a 0.139 g/dL decrease in postpartum hemoglobin (*p* = 0.021). In addition, each doubling of the sFlt-1/PlGF ratio corresponded to a 20.7 mg/dL decrease in maternal fibrinogen concentrations (*p* < 0.001).

Regarding neonatal outcomes, higher sFlt-1/PlGF values were associated with lower neonatal birthweight. Each doubling of the ratio was associated with an approximate 64.6 g reduction in birthweight (*p* = 0.001).

Umbilical artery pH demonstrated a negative trend with increasing sFlt-1/PlGF values; however, this association did not reach statistical significance after adjustment (estimated change −0.0076 per doubling; *p* = 0.070). No statistically significant associations were observed between the sFlt-1/PlGF ratio and neonatal total bilirubin concentrations measured either immediately after birth or on the second day of life.

### 3.4. Quartile-Based Analysis of Outcomes (Q4 vs. Q1)

**Figure 1:**
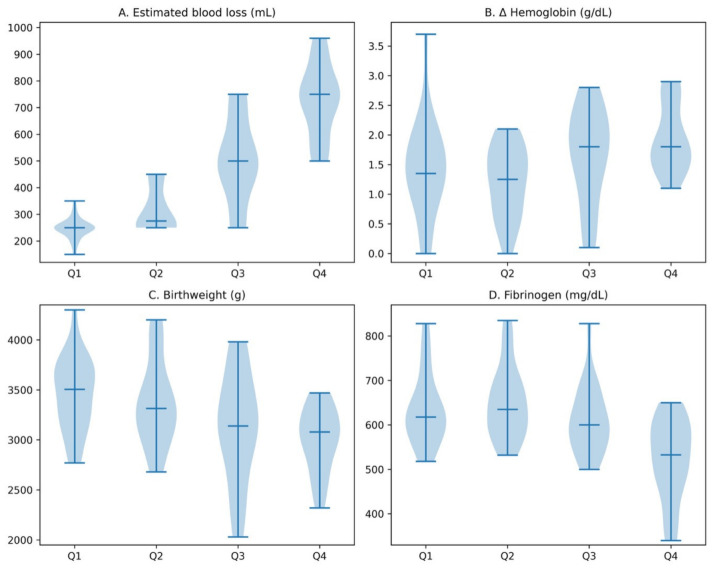
Quartile-based distribution of maternal and neonatal outcomes according to sFlt-1/PlGF values. Violin plots illustrate the distribution and median values of (**A**) estimated blood loss, (**B**) peripartum hemoglobin decrease (ΔHb), (**C**) neonatal birthweight, and (**D**) fibrinogen concentrations across sFlt-1/PlGF quartiles. Quartile boundaries were Q1 ≤ 4.42, Q2 = 4.43–14.46, Q3 = 14.47–34.55, and Q4 > 34.55. Quartiles contained 22, 22, 21, and 22 participants, respectively.

To further characterize the clinical impact of increasing sFlt-1/PlGF values, a quartile-based analysis was performed by comparing women in the highest quartile (Q4) with those in the lowest quartile (Q1). Quartiles were defined based on the distribution of the sFlt-1/PlGF ratio, while outcome differences are presented in original clinical units to facilitate interpretation. All models were adjusted for maternal age, body mass index, parity, and gestational age at delivery. Quartile-based differences in maternal and neonatal outcomes are illustrated in Figure 1.

### 3.5. Maternal Outcomes

Women in the highest sFlt-1/PlGF quartile experienced substantially worse bleeding-related outcomes compared with those in the lowest quartile. Estimated blood loss was significantly higher in Q4, with an adjusted mean difference of approximately +473 mL (*p* < 0.001). Similarly, the peripartum decrease in maternal hemoglobin concentration (ΔHb) was greater in Q4, with an adjusted difference of +0.46 g/dL (*p* = 0.037).

Consistent with these findings, absolute postpartum hemoglobin concentrations were significantly lower in the highest quartile (*p* < 0.001), reflecting the cumulative clinical consequences of increased peripartum blood loss.

Fibrinogen concentrations measured at hospital admission were significantly lower in the highest sFlt-1/PlGF quartile compared with the lowest quartile, with an adjusted difference of −125 mg/dL (*p* < 0.001), which may indicate a reduced hemostatic reserve at higher sFlt-1/PlGF levels.

### 3.6. Neonatal Outcomes

Birthweight was significantly lower among neonates born to women in the highest sFlt-1/PlGF quartile compared with those in the lowest quartile, with an adjusted difference of approximately −428 g (*p* < 0.001). Umbilical artery pH demonstrated a nonsignificant negative trend across quartiles (*p* = 0.070). No statistically significant differences were observed in neonatal total bilirubin concentrations measured immediately after birth or on the second day of life.

### 3.7. Binary and Continuous Outcomes

In quartile-based logistic regression analyses, the sFlt-1/PlGF ratio was not significantly associated with uterine atony, PROM, or umbilical artery pulsatility index above the 75th centile (all *p* > 0.05).

Effect sizes derived from continuous and quartile-based models are presented in Table 3. Continuous analyses use ln-transformed sFlt-1/PlGF and are reported as adjusted changes per doubling of the ratio. Quartile-based analyses compare Q4 vs. Q1 in the original clinical scale.

### 3.8. Exploratory Findings

In exploratory analyses, higher sFlt-1/PlGF values were modestly associated with lower amniotic fluid volume assessed by maximum vertical pocket (Spearman ρ = −0.22, *p* = 0.038). This association was consistent with the overall pattern of subclinical placental dysfunction observed at higher sFlt-1/PlGF levels but was not further explored in multivariable models.

## 4. Discussion

In this study of low-risk term pregnancies, we evaluated the association between the sFlt-1/PlGF ratio and maternal and neonatal outcomes beyond established diagnostic cut-offs. The principal findings were that higher sFlt-1/PlGF ratios were independently associated with greater peripartum blood loss, a larger postpartum decline in hemoglobin concentration, lower maternal fibrinogen levels, and lower neonatal birthweight. These findings suggest that even in uncomplicated term pregnancies, variation in angiogenic balance may reflect subclinical placental dysfunction with clinically relevant maternal and fetal consequences. In contrast, previous studies have primarily focused on high-risk pregnancies, where this ratio has been used to identify complications related to abnormal placental angiogenesis [22,23,24,25].

The sFlt-1/PlGF ratio has been primarily investigated in the context of preeclampsia and fetal growth restriction. In cohorts of women with suspected or confirmed preeclampsia, the sFlt-1/PlGF ratio demonstrated strong diagnostic performance. Saleh et al. reported positive and negative predictive values of 95% and 88%, respectively, using a cutoff value of ≥85 [26]. Consistent with these findings, the PROGNOSIS study by Zeisler et al. showed that an sFlt-1/PlGF ratio ≤38 effectively ruled out the development of preeclampsia within one week, with a negative predictive value of 99.3%, while higher ratios were associated with an increased risk of preeclampsia within four weeks [11]. In contrast, data on the significance of angiogenic markers in low-risk pregnancies have remained limited, and their potential associations with intrapartum and postpartum maternal outcomes have been largely unexplored.

Our findings may indicate an association between increasing sFlt-1/PlGF values and peripartum blood loss. Angiogenic mediators play a key role in vascular development and endothelial stability during pregnancy. Vascular endothelial growth factor (VEGF) regulates vascular growth, remodeling, and endothelial permeability, whereas the anti-angiogenic factor sFlt-1 acts as a circulating antagonist of VEGF and PlGF. Elevated sFlt-1 levels therefore limit the biological activity of pro-angiogenic factors and may contribute to endothelial dysfunction and impaired vascular homeostasis. Consequently, an increased sFlt-1/PlGF ratio may reflect underlying abnormalities in placental vascular development and maternal endothelial regulation. Placental dysfunction and maternal endothelial impairment may theoretically contribute to an increased peripartum bleeding through several mechanisms. Abnormal placental vascularization and altered vascular remodeling may lead to structurally fragile vessels at the maternal–placental interface, while systemic endothelial dysfunction may impair vascular tone and hemostatic responses following placental separation. These mechanisms could potentially facilitate excessive bleeding in the early postpartum period [27]. Recent studies by Rahavendra et al. and Karpova et al. have shown that angiogenic imbalance reflects not only disease severity in hypertensive disorders of pregnancy but is also associated with clinically relevant maternal complications [1,28]. Similarly, Eskild et al. reported consistently higher maternal sFlt-1 concentrations throughout pregnancy in women who later experienced postpartum bleeding (≥500 mL), with significant differences particularly in the second trimester, while PlGF levels did not differ significantly between groups [29].

Consistent with these reports, our findings demonstrate that higher sFlt-1/PlGF ratios are independently linked to greater peripartum blood loss and a more pronounced decline in maternal hemoglobin concentration, supporting a role for placental and endothelial dysfunction in hemorrhagic risk and subsequent postpartum anemia.

Our observation that increasing sFlt-1/PlGF values were associated with lower maternal fibrinogen concentrations suggests a potential interaction between angiogenic imbalance and coagulation pathways. Lower fibrinogen concentrations have been consistently associated with an increased risk of postpartum hemorrhage. Salomon et al. [30] identified fibrinogen levels < 4.5 g/L as an independent risk factor for postpartum hemorrhage (OR 1.86, 95% CI 1.21–2.87), while Charbit et al. [31] reported that fibrinogen concentrations > 4 g/L had a negative predictive value of 79% for severe postpartum hemorrhage.

Clinical data indicate that anti-angiogenic factors such as sFlt-1 can impair endothelial integrity and alter endothelial–platelet interactions, potentially contributing to both increased bleeding risk and consumption of coagulation factors independently of uterine tone. As highlighted by Deshpande et al. and Herraiz et al., angiogenic dysregulation reflects the severity of placental dysfunction and its systemic maternal effects [32,33]. In this context, the association observed in our study between higher sFlt-1/PlGF ratios and lower neonatal birthweight further supports the presence of underlying placental insufficiency. Taken together, these findings may indicate that even subtle disturbances in angiogenic balance may be associated with measurable alterations in maternal hemostasis and fetal growth.

Observations from a retrospective cohort study by Fung et al. demonstrated that elevated sFlt-1/PlGF ratios were associated with adverse neonatal outcomes indicative of chronic placental dysfunction, including small for gestational age, feeding intolerance, prolonged need for parenteral nutrition, respiratory support, and extended hospitalization [34]. In our study, although higher sFlt-1/PlGF values were associated with a tendency toward lower umbilical artery pH, this relationship did not remain significant after adjustment and therefore does not support a clinically relevant effect on acute neonatal acid–base status at birth. Similarly, neonatal bilirubin concentrations were not associated with the sFlt-1/PlGF ratio, suggesting that placental angiogenic dysfunction is unlikely to influence early postnatal bilirubin metabolism. Taken together, these findings support the concept that the impact of sFlt-1/PlGF on acute neonatal biochemical parameters is limited.

This study shifts the focus from diagnostic thresholds to the sFlt-1/PlGF ratio as a continuous marker of angiogenic balance in low-risk pregnancies. Our findings suggest that variation within the physiological range of angiogenic markers may still provide insight into maternal and fetal adaptation in late pregnancy and around delivery.

This study has several limitations that should be acknowledged. First, angiogenic markers were measured late in pregnancy, which precludes assessment of earlier gestational changes in the sFlt-1/PlGF ratio or their temporal dynamics. Second, the study was conducted at a single center and included an ethnically homogeneous population, which may limit the generalizability of the findings to more diverse obstetric populations. In addition, the relatively small sample size did not allow for the identification of clinically meaningful cutoff values for the sFlt-1/PlGF ratio. The modest cohort size may also have limited statistical power to detect weaker associations, particularly for less frequent outcomes; therefore, the possibility of type II error cannot be excluded.

Regression estimates derived from multivariable models in smaller cohorts may be sensitive to model specification and should therefore be interpreted with caution. To reduce the risk of overfitting, the number of covariates included in the models was limited and prespecified based on clinical relevance. Although the models were adjusted for key maternal and obstetric factors, other variables associated with peripartum increased blood loss were not included; therefore, residual confounding cannot be excluded.

Finally, peripartum blood loss was estimated visually using a standardized clinical method rather than objective quantitative tools such as calibrated collection drapes. Visual estimation of blood loss is known to be subject to measurement error and may lead to under- or overestimation of actual blood loss. Importantly, the obstetric staff responsible for estimating blood loss were blinded to biomarker values, which reduces the likelihood of differential measurement bias.

A key strength of the study is the inclusion of women with uncomplicated, term, physiological pregnancies, a population that has been underrepresented in previous research on angiogenic biomarkers. This approach expands the current understanding of sFlt-1/PlGF beyond its established role in pathological pregnancies. Furthermore, the evaluation of both peripartum maternal outcomes and neonatal parameters provides a broader clinical context and may help identify factors relevant to the prevention of adverse perinatal events.

Whilst previous studies have focused on the diagnostic use of the sFlt-1/PlGF ratio in high-risk pregnancies, future prospective studies should explore its role as a continuous marker of peripartum risk in uncomplicated term pregnancies.

These findings support further investigation of the sFlt-1/PlGF ratio as a potential marker of peripartum risk in uncomplicated term pregnancies. However, the present findings are exploratory and do not support clinical implementation or changes in patient management at this stage. Further studies are required to determine whether incorporating angiogenic markers into peripartum risk stratification could improve maternal outcomes beyond established clinical predictors.

## 5. Conclusions

Our results suggest that variability in the sFlt-1/PlGF ratio within uncomplicated pregnancies is associated with greater peripartum blood loss, a more pronounced postpartum decline in maternal hemoglobin levels. Higher sFlt-1/PlGF values were also linked to lower neonatal birthweight and showed a modest association with reduced amniotic fluid volume. Larger prospective studies are warranted to confirm these findings and to determine whether angiogenic markers can improve peripartum risk stratification beyond currently established thresholds.

## Figures and Tables

**Table 1:** Definition of exposure, outcomes, and covariates.

Category	Variable	Definition/Measurement
Exposure	sFlt-1/PlGF ratio	Calculated from maternal serum concentrations of soluble fms-like tyrosine kinase-1 (sFlt-1) and placental growth factor (PlGF) obtained at hospital admission prior to delivery.
Primary outcomes	Estimated blood loss (mL)	Clinically estimated maternal blood loss recorded at delivery, assessed by the attending obstetric team using a standardized Gauze Visual Analogue method [20]. The estimation was based on visual assessment of blood-soaked gauzes and other absorbent materials used during delivery, in accordance with routine clinical practice. Blood loss was independently assessed by two physicians involved in the delivery care, and the final value was determined by consensus. The clinical staff responsible for blood loss estimation were blinded to maternal angiogenic biomarker values.
Secondary outcomes	Platelet count (×10^3^/µL)	Measured before and after delivery.
	Activated partial thromboplastin time (APTT)	Measured at hospital admission.
	International normalized ratio (INR)	Measured at hospital admission.
	Δ Hemoglobin (g/dL)	Difference between pre-delivery and post-delivery maternal hemoglobin concentrations.
	Fibrinogen (mg/dL)	Measured at hospital admission.
	Umbilical artery pH	Umbilical artery pH measured immediately after delivery.
	Birthweight (g)	Neonatal birthweight measured at delivery.
	Total bilirubin (mg/dL)	Neonatal total bilirubin concentration measured after birth, and on the second day of life.
	Uterine atony	Clinically diagnosed intrapartum uterine hypotonia requiring standard obstetric management.
	Amniotic fluid volume (MVP)	Measured at hospital admission.
	Premature rupture of membranes	Rupture of membranes prior to the onset of labor.
	Umbilical artery PI > 75th centile	Measured at hospital admission.
Covariates	Maternal age (years)	Age at delivery.
	Body mass index (kg/m^2^)	Body mass index calculated at admission.
	Parity	Nulliparous or multiparous.
	Gestational age at delivery (weeks)	Gestational age at the time of delivery.

**Table 2:** (**a**) Maternal and angiogenic characteristics of the study cohort stratified by mode of delivery. (**b**) Neonatal outcomes stratified by mode of delivery.

**(a)**			
**Characteristic**	**Overall Cohort** **(*N* = 87)**	**Vaginal Delivery** **(*N* = 41)**	**Cesarean Section** **(*N* = 46)**
sFlt-1 (pg/mL)	1472 (668–3177)	1585 (641–3190)	1126 (425–2357)
PlGF (pg/mL)	100 (48–260)	103 (52–259)	100 (44–263)
sFlt-1/PlGF ratio	14.46 (4.42–34.55)	16.50 (5.88–30.96)	10.65 (3.12–37.83)
Maternal age (years)	32 (30–35)	33 (31–35)	31.5 (29.3–35.8)
Body mass index (kg/m^2^)	28.41 (24.89–30.58)	27.82 (24.57–29.39)	28.85 (25.12–32.92)
Gestational age at delivery (weeks)	39 (38–40)	40 (39–41)	38 (38–39)
Estimated blood loss (mL)	450 (250–625)	250 (250–550)	450 (250–725)
Hemoglobin before delivery (g/dL)	12.5 (12.05–13.3)	12.6 (12.1–13.4)	12.45 (11.93–13.2)
Hemoglobin after delivery (g/dL)	11.0 (10.35–11.6)	11.0 (10.4–11.6)	10.9 (10.23–11.55)
Change in hemoglobin (ΔHb) (g/dL)	1.6 (1.2–1.95)	1.5 (1.1–1.9)	1.65 (1.23–2.08)
Fibrinogen (mg/dL)	600 (548–661)	600 (548–682)	600 (559–633)
APTT (s)	27.5 (26.2–28.95)	28.0 (26.3–28.7)	27.2 (26.2–29.3)
INR (N)	0.96 (0.93–1.00)	0.96 (0.94–1.00)	0.96 (0.93–1.00)
**(b)**			
**Characteristic**	**Overall Cohort** **(*N* = 87)**	**Vaginal Delivery** **(*N* = 41)**	**Cesarean Section** **(*N* = 46)**
Birthweight (g)	3240 (3000–3500)	3250 (3060–3680)	3190 (2942–3395)
Umbilical artery pH	7.33 (7.27–7.35)	7.33 (7.26–7.35)	7.33 (7.30–7.35)
Total bilirubin (mg/dL)	5.10 (4.15–6.40)	5.5 (4.6–6.8)	4.85 (3.79–6.2)
Total bilirubin—day 2 (mg/dL)	7.90 (6.15–9.60)	9.3 (7.5–10.3)	7.45 (5.55–8.4)

Data are presented as median (interquartile range).

**Table 3:** Effect sizes of sFlt-1/PlGF for maternal and neonatal outcomes.

Outcome	Continuous Analysis	95% CI	*p*-Value	Quartile Analysis	95% CI	*p*-Value
Blood loss	+78.0 mL	42.1 to 113.9	<0.001	+473 mL	260 to 690	<0.001
Δ Hemoglobin	+0.078 g/dL	0.008 to 0.148	0.030	+0.46 g/dL	0.03 to 0.89	0.037
Fibrinogen	−20.7 mg/dL	−30.5 to −10.9	<0.001	−125 mg/dL	−190 to −60	<0.001
Birthweight	−64.6 g	−102.0 to −27.2	0.001	−428 g	−650 to −210	<0.001

## Data Availability

The original contributions presented in the study are included in the article; further inquiries can be directed to the corresponding author.
